# Emerging Treatments for Malignant Pleural Mesothelioma: Where Are We Heading?

**DOI:** 10.3389/fonc.2020.00343

**Published:** 2020-03-12

**Authors:** Luca Cantini, Raffit Hassan, Daniel H. Sterman, Joachim G. J. V. Aerts

**Affiliations:** ^1^Department of Pulmonary Medicine, Erasmus MC, Rotterdam, Netherlands; ^2^Erasmus Cancer Institute, Erasmus MC, Rotterdam, Netherlands; ^3^Clinical Oncology, Università Politecnica delle Marche, AOU Ospedali Riuniti Ancona, Ancona, Italy; ^4^Thoracic and GI Malignancies Branch, Center for Cancer Research (CCR), National Cancer Institute (NCI), National Institutes of Health (NIH), Bethesda, MD, United States; ^5^Division of Pulmonary, Critical Care, and Sleep Medicine, New York University (NYU) School of Medicine/NYU Langone Medical Center, New York, NY, United States

**Keywords:** Malignant mesothelioma, checkpoint inhibitors, immunotherapy, tumor-treating fields, dendritic cell therapy, mesothelin, anti-angiogenic, targeted therapy

## Abstract

Malignant pleural mesothelioma (MPM) is an uncommon but aggressive and treatment resistant neoplasm with low survival rates. In the last years we assisted to an exponential growth in the appreciation of mesothelioma pathobiology, leading several new treatments to be investigated both in the early stage of the disease and in the advanced setting. In particular, expectations are now high that immunotherapy will have a leading role in the next years. However, caution is required as results from phase II studies in MPM were often not replicated in larger, randomized, phase III trials. In this review, we describe the most promising emerging therapies for the treatment of MPM, discussing the biological rationale underlying their development as well as the issues surrounding clinical trial design and proper selection of patients for every treatment.

## Introduction

Malignant pleural mesothelioma (MPM) is an uncommon and highly lethal cancer. The annual incidence of MPM ranges between 10 cases per million to 29 cases per million depending on the country and, because of the long latency period, the peak is expected in the 2020s (1) in high-income countries. In addition, according to WHO prediction (2), developing countries where asbestos is still used, are likely to face a new epidemic of asbestos-related diseases, including MPM.

MPM pathogenesis is peculiar, as the direct causal relationship between exposure to airborne asbestos particles and the development of MPM is well established (3). The chronic exposure to asbestos fibers, which may enter the lung periphery and the pleura, leads to chronic inflammation of the mesothelium which sustains the carcinogenic processes (4). Individuals with germline BRCA1 associated protein-1 (BAP1) mutations may be predisposed to MPM, since they may develop it without any apparent asbestos exposure (5). Recent biological and preclinical studies provided further insights into MPM carcinogenesis, revealing the importance of tumor suppressor gene inactivation, through several mechanisms (single nucleotide variants (SNVs), copy number losses, gene fusions, and splicing alterations). Tumor suppressor genes highly altered are cyclin-dependent kinase inhibitor 2A (CDKN2A, 60% of the cases), BAP1 (60% of the cases also in sporadic MPM), and neurofibromin 2 (NF2, 75% of the cases) (6–9).

The chronic inflammatory response to asbestos involved in the pathogenesis of MPM also causes a unique tumor environment. This microenvironment is mainly composed of immunosuppressive cells [regulatory T cells, macrophages and myeloid-derived suppressor cells (MDSCs)] and the number of these cells as determined by immunohistochemistry (IHC) represents a negative prognostic factor (10, 11). On the other hand, immune-activating responses, such as the presence of CD8^+^ T cells, are correlated with better outcome, although such links with prognosis are less important when compared with other cancer entities which are more immunogenic than MPM (12).

The management of MPM is complex and outcomes remain poor. For patients with early stage MPM the role of radical surgery is still a matter of debate and it should be considered only as part of a multimodal treatment (i.e., surgery combined with chemotherapy, radiotherapy, or both). Looking at unresectable MPM, no major breakthroughs have been made since the approval of antifolate and platinum combination chemotherapy (13, 14). Median overall survival (OS) time with standard first-line options is about 13 months, with the best outcome for the epithelioid MPM subtype (14). Second-line treatment scenario is even more disappointing. With the only exception of a repeated course of pemetrexed-based chemotherapy for previously responsive patients (15), limited options are available for relapsed MPM and new treatments are urgently needed.

**Figure 1:**
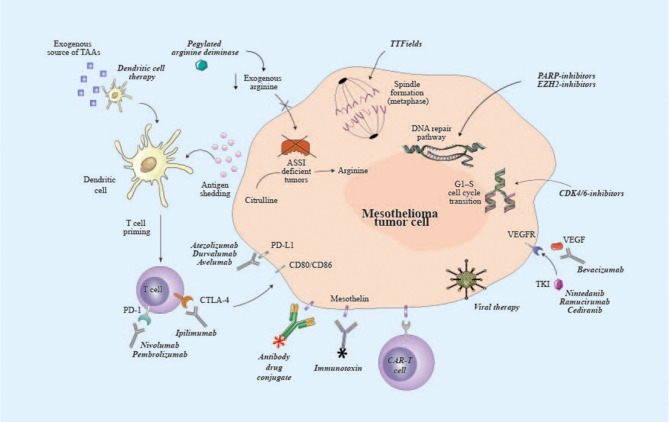
Potential targets of emerging therapies for malignant pleural mesothelioma. ASSI, argininosuccinate synthase I; CAR, chimeric antigen receptor; CD80, cluster of differentiation 80; CD86, cluster of differentiation 86; CDK4/6, cyclin-dependent kinase 4/6; CTLA-4, cytotoxic T lymphocyte associated protein-4; EZH2, enhancer of zeste homolog 2; PARP, poly ADP ribose polymerase; PD-1, programmed cell death-1; PD-L1, programmed death ligand-1; TAAs, tumor-associated antigens; TKI, tyrosine kinase inhibitor; TTF, tumor-treating fields; VEGFR, vascular endothelial growth factor receptor.

Steps have been made toward a best appreciation of mesothelioma biology and have been essential to identify novel molecular therapeutic targets, representing the rationale for testing multiple targeted therapies in MPM (Table 1). Nevertheless, the potential to improve the potency and the specificity of the immune system, along with recent successes in other thoracic tumors, have attracted a growing interest in cancer immunotherapy. Continue efforts are necessary to further deepen our understanding of mesothelioma, taking into account biological and temporal heterogeneity of the disease in order to finally optimize the development of new treatment options in the context of well-designed clinical trials (Figure 1).

**Table 1:** Ongoing trials in malignant pleural mesothelioma patients (source: ClinicalTrials.gov).

**Class**	**Treatment**	**Trial name/Identifier**	**Phase**	**Setting/Line of treatment**	**Single agent/Combined therapy**	**Estimated enrollment**	**Notes**
Surgery	eP/D	NCT02040272 (MARS2)	III	Surgically resectable	Standard neoadjuvant chemotherapy before surgery	328	Multicentre randomized trial comparing eP/D vs. no surgery
	eP/D - chemotherapy	NCT02436733	II	Surgically resectable	Neoadjuvant or adjuvant chemotherapy	64	Chemotherapy before or after P/D in patients with early stage MPM
Radiotherapy	Accelerated hypofractionated radiotherapy with tomotherapy	NCT03269227	I	Adjuvant (after eP/D)	N/A	30	
	Hemitoracic intensity modulated radiation therapy (IMPRINT)	NCT00715611	II	Adjuvant	Adjuvant chemotherapy	81	After enrolling 45 patients, hemithoracic IMPRINT was safe and had an acceptable rate of pneumonia
	Short neoadjuvant hemithoracic intensity-modulated radiation therapy	NCT00797719	I	Neoadjuvant	Adjuvant chemotherapy (+/-)	100	
Chemotherapy	Mithramycin (continuous 24-hours infusion)	NCT02859415	I/II	Relapsed	Single agent	100	Mithramycin is an antineoplastic antibiotic that inhibits cancer stem cell signaling
Antiangiogenic agents	Nintedanib	NCT02863055	II	Maintenance treatment after chemotherapy	Single agent	116	
PARP inhibitors	Olaparib	NCT03531840	II	Relapsed	Single agent	40	Recruitment is not limited to patients with germline/somatic mutations in DNA repair genes
	Niraparib	NCT03207347	II	Relapsed	Single agent	57	
EZH2 inhibitors	Tazemetostat	NCT02875548	Extension (rollover)	Relapsed	N/A	300	In multiple solid tumors
Base-excision repair inhibitors	TRC-102	NCT02535312	I/II	First line/Relapsed	Cisplatin and pemetrexed or only pemetrexed	58	
PI3K inhibitors	IPI-549	NCT02637531	I	Relapsed	Nivolumab (+/-)	220	In multiple solid tumors
FAK inhibitors	Defactinib	NCT02004028	Window-of-opportunity	Neoadjuvant	Single agent	38	
		NCT02758587	I/II	Relapsed	Pembrolizumab	59	
	APG-2449	NCT03917043	I	Relapsed	Single agent	40	APG-2449 is a novel, oral, multi-targeted tyrosine kinase inhibitor, which inhibits FAK, ALK, and ROS1
BCR/ABL pathway	Bosutinib	NCT03023319	I	N/A	Pemetrexed	24	In multiple solid tumors
Arginine deprivation	ADI PEG 20	NCT02709512 (ATOMIC)	II/III	First line	Cisplatin and pemetrexed	386	Double-blind, randomized (standard chemotherapy in the control group); only patients with biphasic or sarcomatoid histology are eligible; ASS1-deficiency is not required for study entry
Arginase inhibitors	INCB001158	NCT02903914	I	Relapsed	Pembrolizumab	424	In multiple solid tumors
Anti-CD30	Brentuximab vedotin	NCT03007030	II	Any line	Single agent	55	CD30 positive MPM
MDM2 antagonists (p53 pathway)	ASTX295	NCT03975387	I	Relapsed	Single agent	135	In multiple solid tumors (p53 wild type)
DR5 agonists	INBRX-109	NCT03715933	I	Relapsed	Single agent	80	INBRX-109 is a multivalent agonist of DR5
Tie2 inhibitors	Rebastinib (DCC-2036)	NCT03717415	I	First line/Relapsed	Carboplatin	117	Rebastinib acts on Tie2, a tyrosine kinase receptor that is expressed on endothelial cells and pro-tumoral macrophages
Immune check-point inhibitors	Pembrolizumab	NCT02707666	Window-of-opportunity	Neoadjuvant	Adjuvant pemetrexed and cisplatin	15	
		NCT02784171	II/III	First line	Cisplatin and pemetrexed	126	Randomized trial with both cisplatin/pemetrexed and pembrolizumab alone (only in the phase II part) as active comparators
		NCT02959463	I	Adjuvant to radiotherapy	N/A	24	Primary goal is to determine the safety and tolerability of pembrolizumab administered after radiation therapy in patients with MPM who have not undergone EPP
		NCT03393858	II	Relapsed	DC-CIK immunotherapy combined with hyperthermia	40	
		NCT02628067 (KEYNOTE-158)	II	Relapsed	Single agent	1350	A trial of pembrolizumab (MK-3475) to evaluate predictive biomarkers in advanced cancers
	Nivolumab	NCT03063450 (CONFIRM)	III	Relapsed	Single agent	336	Double-blind, placebo controlled
		NCT03502746	II	Relapsed	Ramucirumab	35	
		NCT02834013	II	Relapsed	Ipilimumab	707	Anti-CTLA-4 and Anti-PD-1 combination in rare tumors
	MEDI4736	NCT02592551	Window-of-opportunity	Neoadjuvant	Tremelimumab (only 8 patients)	20	
	Atezolizumab	NCT03762018 (BEAT-meso)	III	First line	Bevacizumab and standard chemotherapy	320	Open-label, randomized (bevacizumab plus standard chemotherapy in the control group)
		NCT03074513	II	Relapsed	Bevacizumab	160	
		NCT03228537	I	Neoadjuvant	Cisplatin and Pemetrexed	28	Within 90 days after completion of surgery patients receive atezolizumab for up to 1 year
	Avelumab	NCT03399552	I	Adjuvant to radiotherapy (stereotactic body radiation therapy)	N/A	27	
	INCMGA00012	NCT03920839	I	First line	Cisplatin and pemetrexed	98	INCMGA00012 is a humanized IgG4 monoclonal antibody that targets human PD-1 and lacks antibody dependent cell-mediated cytotoxicity directed against effector lymphocytes
	XmAb20717	NCT03517488	I	Relapsed	Single agent	87	Phase I trial assessing the safety and tolerability of XmAb20717, a bispecific antibody that simultaneously targets immune checkpoint receptors PD-1 and CTLA-4, in multiple tumors
	Cosibelimab	NCT03212404	I	Relapsed	Single agent	500	In multiple solid tumors; CK-301 (cosibelimab) is a fully human monoclonal IgG1 antibody against PD-L1
	ABBV-181	NCT03000257	I	N/A	Single agent	221	In multiple solid tumors; ABBV-181 is an anti-PD1 monoclonal antibody
	TIM-3 inhibitor (INCAGN02390)	NCT03652077	I	Relapsed	Single agent	41	In multiple solid tumors
	LAG-3 inhibitor (INCAGN02385)	NCT03538028	I	Relapsed	Single agent	40	In multiple solid tumors
	GITR agonist (INCAGN01876)	NCT03126110	I/II	Relapsed	Nivolumab/Ipilimumab	285	In multiple solid tumors
	OX40 agonist (ABBV-368)	NCT03071757	I	Relapsed	Single agent/combination with anti-PD1 therapy	170	In multiple solid tumors
Mesothelin targeted therapy	Immunotoxin LMB-100	NCT03644550	II	Relapsed	Pembrolizumab	38	
		NCT04034238	I	Relapsed	Tofacitinib (inhibitor of Janus kinases)	45	
	Anetumab ravatansine	NCT03126630	I/II	Relapsed	Pembrolizumab	134	Open-label, randomized but not comparative (pembrolizumab alone in the non-experimental arm)
		NCT03926143	Extension (rollover)	Relapsed	N/A	20	
	Thorium-227 labeled antibody-chelator conjugate (BAY2287411)	NCT03507452	I	Relapsed	N/A	228	All tumors known to express mesothelin are eligible
Vaccines	Galinpepimut-S	NCT04040231	I	Relapsed	Nivolumab	10	
	Dendritic cell therapy (Mesopher)	NCT03610360 (DENIM)	II/III	Maintenance treatment after chemotherapy	Single agent	230	Dendritic cells are loaded with allogeneic tumor cell lysate (PheraLys)
		NCT02649829	I	Neoadjuvant	Standard concomitant chemotherapy and eP/D afterwards (in case of resectable disease)	20	Dendritic cells are loaded with the tumor antigen WT1
Adoptive cell therapy	iCasp9M28z CAR-T cells (targeting mesothelin)	NCT02414269	I	Relapsed	Cyclophosphamide prior to infusion +/- Pembrolizumab after infusion	66	After treating 20 patients, intrapleurally administered mesothelin-targeted CAR T cells were safe with encouraging antitumor activity
	TC-210 CAR-T cells (targeting mesothelin)	NCT03907852	I/II	Relapsed	Cyclophosphamide and fludarabine before treatment as lymphodepleting agents	70	
	CAR-T cells (targeting mesothelin)	NCT03638206	I	N/A	Cyclophosphamide and fludarabine	73	In multiple solid tumors
	TILs	NCT02414945	I/II	N/A	Cyclophosphamide and Fludarabine before treatment, low-dose IL-2 after cell infusion	10	
		NCT03935893	I	Relapsed	Cyclophosphamide and fludarabine	10	
Virotherapy	Intrapleural adenonovirus-deliveres interferon alpha-2b (rAd-IFN)	NCT03710876 (INFINITE)	III	Relapsed	Celecoxib and gemcitabine	300	Open-label, randomized with control group receiving only oral celecoxib plus intravenous gemcitabine
Other intrapleural therapies	Intrapleural Cryotherapy	NCT02464904	I	Neoadjuvant	N/A	15	
	Hyperthermic intraoperative chemotherapy (with pemetrexed and cisplatin)	NCT02838745	I	Adjuvant	N/A	36	
	Intracavitary cisplatin-fibrin localized chemotherapy	NCT01644994	I/II	Adjuvant	N/A	54	
	Intraoperative porfimer sodium -mediated photodynamic therapy	NCT02153229	II	Adjuvant	N/A	102	Open-label, randomized

*N/A, data not available; ALK, anaplastic lymphoma kinase; ASSI, argininosuccinate synthase I; CAR, chimeric antigen receptor; CD30, cluster of differentiation 30; CTLA-4, cytotoxic T lymphocyte associated protein-4; DC-CIK, autologous dendritic cells-cytokine induced killer cell; DR5, death receptor 5; eP/D, extended pleurectomy and decortication; EPP, extrapleural pneumonectomy; EZH2, enhancer of zeste homolog 2; FAK, focal adhesion kinase; IgG, immunoglobulin G; MDM2, murine double minute 2; MPM, malignant pleural mesothelioma; PARP, poly ADP ribose polymerase; PD-1, programmed cell death-1; PI3K, phosphoinositide 3-kinase; ROS1, ROS proto-oncogene 1; TIE2, tyrosine kinase with immunoglobulin-like and EGF-like domains 1; WT1, Wilms' tumor*.

In this review, we describe last emerging therapies for mesothelioma, discussing the current status of knowledge in mesothelioma genetics and immune-biology, as well as the issues surrounding the conduction of high-quality trials in MPM and the selection of best patients for different treatments.

## Neoadjuvant/Adjuvant Setting

Due to the anatomy, microscopically radical (R0) resection is not achievable in mesothelioma surgery and the goal of mesothelioma surgery is macroscopic complete resection (R1). Surgery alone is not curative; it is usually performed with chemotherapy and/or radiation therapy and reserved to a subset of patients with early tumor stage, epithelioid histology and good performance status.

Therapeutic surgery in mesothelioma has historically involved either an extended pleurectomy-decortication (eP/D) or an extrapleural pneumonectomy (EPP) (16, 17). eP/D has been proven to offer better results in the context of multimodality treatment (18, 19), and although the benefit of systemic therapy has been shown only in the advanced/unresectable disease, it is common practice to give four cycles of cisplatin or carboplatin with pemetrexed as adjuvant or neoadjuvant therapy. Two on-going trials, MARS 2 (NCT02040272) and EORTC1205-LCG (NCT02436733), are currently evaluating the usefulness, the feasibility and the best timing for the combined approach of surgery and chemotherapy.

In order to improve local control and ideally survival, radiotherapy can be given. New approaches of radical hemithoracic radiation using intensity-modulated techniques are being tested. Rimner et al. showed that hemithoracic intensity-modulated pleural radiation therapy (IMPRINT) after chemotherapy and P/D was safe in 27 MPM patients as part of a multimodality lung-sparing treatment, with an acceptable rate of radiation pneumonitis (20). Larger clinical trials are awaited to confirm the effectiveness of this approach.

Recently, intrapleural therapies have been reported with the aim of improving loco-regional control of the disease by spreading drugs directly on the tumor surface. Several techniques with different rationale have been used with promising results: hypertermic intrapleural chemotherapy, photodynamic therapy (PDT), intrapleural immunotherapies [interferons (IFNs) and interleukin-2 (IL-2)], and gene therapy (21). However, available evidences are mainly based on retrospective, small and single-institution studies and controlled randomized trials are required.

If given as neoadjuvant therapy, novel agents should have the ability to induce tumor shrinkage, increasing the possibility of a complete microscopic resection and ultimately prolonging overall survival while maintaining a good safety profile. Designing studies in this setting remains a challenging effort that requires multidisciplinary involvement (22). Nevertheless, the neoadjuvant setting provides the unique possibility to conduct translational research in the context of window-of-opportunity trials, acquiring valuable information from blood and tissue collection. For example, the focal adhesion kinase (FAK)-inhibitor defactinib showed immunomodulatory effects when administered pre-operatively in a phase II window of opportunity trial (23) with a good tolerability profile, an objective response rate of 13% and 67% of stable disease, thus not altering resectability or mortality compared to historical controls. Final trial data are expected for 2020.

This approach has also paved the way for testing the properties of immune check-point inhibitors (CIs). There are several ongoing neoadjuvant trials which aim to assess the immunomodulatory and pharmacodynamics effect of CIs, as monotherapy (NCT02707666), as combination of anti-cytotoxic T-lymphocyte-associated protein 4 (CTLA4) and anti-programmed cell death protein (PD-1) agents (NCT02592551, NCT03918252) and as combination of anti-programmed death-ligand 1 (PD-L1) with standard chemotherapy (NCT03228537).

By assessing translational surrogates of response, these trials may represent an opportunity to look into predictive biomarkers, improving selection of candidates to CIs-treatment.

CIs are also tested in the adjuvant setting (NCT02707666). From an immunological perspective, the main goal of combining surgery with adjuvant CIs is to reduce tumor induced immunosuppression (24). Increased tumor size correlates with major immune suppression and surgically shrinking tumor size may potentially reduce immune inhibition and T-cell exhaustion (25).

Another approach to increase immune activation in the adjuvant setting is represented by vaccines, either protein, bacteria or cell-based. An adjuvant Wilms tumor 1 (WT1) vaccine (galinpepimut-S), given with granulocyte-macrophage colony-stimulating factor (GM-CSF) and an immunologic adjuvant called montanide ISA 51 UFCH in MPM patients whose tumors expressed WT1 at IHC, had completed combined multimodality therapy and had no evidence of disease, showed a median progression-free survival (PFS) of 10.1 months (95% CI 5.5–20.8 months) and a median OS of 22.8 months (95% CI 9.1–37.6 months) with a favorable safety profile (26). Galinpepimut-S is currently being tested in the advanced setting combined with CI-treatment (NCT04040231).

In peritoneal mesothelioma, the feasibility of administering dendritic cells pulsed with an allogenic tumor cell lysate after cytoreductive surgery and hyperthermic intraperitoneal chemotherapy (HIPEC) is being assessed in the ongoing MESOPEC trial (NTR7060) (27). Secondary objectives of the study are to assess the safety of dendritic cells and determine whether this adjuvant treatment may induce a specific immunological response against the tumor (27). Pre-clinical evidences showed that dendritic cell therapy leads to better outcome when dendritic cells are injected in murine models with lower tumor volume (28, 29). An efficient immune response is hampered by cytokines and regulatory T-cells induced by mesothelioma cells, showing that a low tumor load correlates with a better functioning immune system and higher anti-tumor responses. Giving dendritic cell therapy after surgically reducing tumor load might therefore improve response to therapy and clinical outcome.

To date, despite the neoadjuvant/adjuvant treatment represents a promising setting to test new therapeutic strategies, the global level of evidence is quite low and international guidelines (30) do not recommend either neoadjuvant or adjuvant radiotherapy/chemotherapy as standard options for resectable MPM.

## Unresectable Mesothelioma

### Chemotherapy

There is no approved maintenance treatment for MPM patients who did not progress after first-line chemotherapy. NVALT19 was an open label, multicentric, randomized phase II trial, in which patients were assigned 1:1 to gemcitabine (1,250 mg/m2 day 1 and 8 of 3 weekly schedule) or best supportive care (BSC) after 4-6 cycles of first-line platinum-pemetrexed without progression. Data presented at the last ESMO conference showed an improvement in PFS (median 6.2 months vs. 3.2 months in the BSC arm [hazard ratio (HR) 0.42 (95% CI 0.28-0.63), *p* < 0.0001)], at the cost of an increased yet manageable toxicity (57% of patients experienced grade 3–4 adverse events vs. 13% in the BSC-arm, with neutropenia, nausea and lung infection being the most frequent) (31). Since post-study treatments and OS data were not reported, the reported improvement in PFS could be simply due to an anticipation of second-line therapy.

Lurbinectedin is a new molecule that binds to the DNA minor groove in regulatory regions, inhibiting the function of oncogenic transcription factors. It also modulates the transcriptional program of monocytes and TAMs, hampering cytokine production (32). Investigator tested the role of lurbinectedin in the context of relapsed MPM, where no approved therapy exists. Recent data from the SAKK 17/16 multi-center, single-arm phase II trial, showed activity of lurbinectedin. Median PFS and median OS were 4.1 months (95% CI 2.6-5.5) and 11.9 months (95% CI 9.2–14.7), respectively. Lurbinectedin also worked independently of histology or prior immunotherapy (32).

These data support evaluation of the both gemcitabine as switch maintenance and lurbinectedin as second-line strategy in larger, randomized, phase III trials.

The NovoTTF-100L represents another approach that has been recently investigated to improve the efficacy of chemotherapy. NovoTTF-100L is a portable Tumor Treating Fields (TTFields) delivery system. TTFields represent a non-invasive, regional treatment modality by which alternating electric fields (at a frequency of 150 kHz) are continuously administer to the local site to arrest tumor cancer cell division. In human mesothelioma cell cultures, combining TTFields with cisplatin or pemetrexed led to reduction in cell count, induction of apoptosis and reduced clonogenic potential (33). These alternating electric fields act by disrupting spindle formation during metaphase and blocking the localization of intracellular organelles during telophase.

Based on the results of the prospective, single-arm, phase II STELLAR trial, the NovoTTF-100L System was approved by U.S. FDA in combination with pemetrexed plus platinum-based chemotherapy for the first-line treatment of unresectable locally advanced or metastatic MPM. NovoTTF-100L was approved under Humanitarian Device Exemption, an approval process guaranteed by the U.S. FDA which, taking into consideration the urgent need to identify more effective treatments for rare disease (such as MPM), allows medical devices to be marketed without requiring evidence of effectiveness.

However, the STELLAR trial raised several issues that need to be addressed before implementing this strategy into daily practice. The 80 patients enrolled in the STELLAR trial (34) had a median OS of 18.2 months (95% CI 12.1-25.8), with 40.3% of partial responses and 97.2% of them obtaining a clinical benefit. Response rates were similar to the ones with standard chemotherapy but lasted longer by adding TTFields (median response duration was 5.7 months, ranging from 1.4 to 13 months). The rate of serious systemic adverse events remained the same when NovoTTF-100L was added to chemotherapy (either pemetrexed plus cisplatin or pemetrexed plus carboplatin, according to investigator choice). Expected TTFields-related skin toxicity was reported in 66% (53 patients) with only 5% of grade 3 skin toxicity. These results should be considered in context of the randomized phase III MAPS trial (35), in which bevacizumab added to pemetrexed and cisplatin significantly improved median OS compared to pemetrexed plus cisplatin alone (median OS 18.8 vs. 16.1 months, HR 0.77, *p* = 0.0167). The control arm of this trial performed 4 months better than the historical cohort analyzed by Ceresoli et al.—the landmark study by Vogelzang et al.—(14) and should be considered while discussing STELLAR data. Also PFS (7.6 months) and response (40%) were similar when compared to control groups in the MAPS and the recent LUME-meso trials (36). This fact, together with the potential sampling bias in single-arm studies and the effect of subsequent therapies, limits the interpretation of STELLAR data.

To date, TTFields represent one of many empirical approaches to MMP and further investigation of this approach in randomized trials is strongly encouraged.

### Anti-angiogenic Agents

Activation of the vascular endothelial growth factor (VEGF) pathway, via its tyrosine kinase receptors, is crucial for mesothelioma cells growth (37), thus representing a rationale for antiangiogenic treatments in this neoplasm.

The addition of bevacizumab to pemetrexed and cisplatin chemotherapy as first-line treatment with bevacizumab maintenance therapy in patients who did not progress showed improved overall survival. However, bevacizumab remains currently unlicensed in this setting since the MAPS trial was not a registration trial (35). Moreover, results of Bevacizumab [an anti-VEGF monoclonal antibody (mAb)] as first-line option in combination with chemotherapy were not confirmed by other anti-angiogenic agents, such as the tyrosine-kinase inhibitors (TKIs) axitinib (an anti-VEGFR TKI), sorafenib (anti-VEGFR2/3, platelet-derived growth factor receptor (PDGFR) and rapidly accelerated fibrosarcoma (RAF)/c-KIT), or imatinib mesylate (targeting BCR-ABL, c-KIT, and PDGFR) (38–41).

Since the benefit in the phase 2 trial (*n* = 87 patients) (42) was higher in epithelioid MPM than in non-epithelioid subtypes, the multi-targeted anti-angiogenic kinase inhibitor, nintedanib (targeting VEGFR 1–3, PDGFR α or β, fibroblast growth factor receptor (FGFR) 1–3, SRC and ABL kinases pathways) was tested in conjunction with first-line cisplatin plus pemetrexed in a randomized phase III trial vs. placebo only in patients with epithelioid histology. However, among the 458 randomized patients, the previous phase II efficacy findings were not confirmed and PFS did not differ between the nintedanib group (median 6.8 months [95% CI 6.1–7.0)] and the placebo group (7.0 months (95% CI 6.7–7.2); HR 1.01 (95% CI 0.79–1.30), *p* = 0.91). The interim analysis of OS also showed no difference between groups (36).

Nintedanib is also being currently investigated as only maintenance treatment for patients non-progressive after first line chemotherapy (NCT02863055).

Cediranib, a VEGFR and PDGFR inhibitor, added to first-line platinum-based chemotherapy, improved PFS in a randomized phase II trial (43). Primary end-point of the trial was to detect a PFS difference (by RECIST version 1.1) at the 1-sided 0.10 level and it was met. PFS was significantly higher in MPM patients who received cisplatin-pemetrexed chemotherapy with cediranib followed by maintenance cediranib, compared to the ones receiving cisplatin-pemetrexed with placebo. HR was 0.69 (median PFS 7.2 vs. 5.6 months, *p* = 0.096). However, PFS was not different by modified RECIST and no significant difference in OS was reported. As with bevacizumab, cediranib is not approved as first-line treatment combined with chemotherapy.

Ramucirumab is a monoclonal antibody that binds the extracellular domain of human VEGFR-2. Due to VEGF-R2 expression on macrophages, ramucirumab also inhibits macrophages and their infiltration into mesothelioma microenvironment, thereby decreasing tumor growth and proliferation (44). One-hundred sixty-four patients are planned to be randomized in a multicenter, double-blind, placebo-controlled phase II trial comparing gemcitabine with or without ramucirumab in the second-line setting [NCT03560973 (RAMES)], whose completion is expected for 2020.

### Targeted Therapies

New studies have recently provided a comprehensive genomic profiling of mesothelioma. Genomic analysis may help in detecting actionable alterations and developing more tailored and effective therapies for MPM patients (6). Tumor suppressor inactivation (loss-of-function) represents one of the most frequent mutational events in this tumor. In addition, multiple studies have pointed out frequent copy gains and copy losses involving different portions of the genome (6, 7, 45–48).

Carriers of inherited loss-of-function mutations in BAP1 are predisposed to mesothelioma (5, 45, 49, 50). BAP1 encodes a deubiquitinase enzyme, a member of the ubiquitin carboxy (C)-terminal hydrolase (UCH) family, involved in different cellular pathways among which the cell cycle, cellular differentiation, cell death, metabolism, and the DNA damage response (51). In particular, BAP1 is thought to bind to the breast cancer type 1 susceptibility protein (BRCA1) and the BRCA1-associated RING domain protein 1 (BARD1) and enhance their tumor suppressor function (52). Besides germline mutations, recent analysis of the BAP1 locus by targeted next-generation sequencing identified homozygous inactivating mutations in approximately 60% of patients (53). This implies that the role of BAP1 in defective DNA repair and homologous recombination might be therapeutically exploited in a large number of MPM.

In a recent paper, among 385 patients treated with platinum chemotherapy, median OS was increased for MPM patients who had inherited mutations in DNA repair and/or other tumor suppressor genes (54). This is consistent with what already observed in ovarian and breast cancer patients with inherited mutations in BRCA1 and BRCA2 (55–58). Conversely, BAP1 mutant mesothelioma cell lines resulted significantly less sensitive than BAP1 wild type cells to gemcitabine (59). In addition, the role of somatic BAP1 expression in MPM patients receiving chemotherapy still represents a matter of debate, with retrospective studies showing contradictory evidences (60, 61).

By inducing synthetic lethality of alternate DNA repair pathways, poly-ADP ribose polymerase (PARP) inhibitors have proved to be able to cause cell death in cell lines with loss of function of BAP1. This observation suggests that patients with mutations in BAP1 and in DNA repair genes might also benefit from treatment with PARP inhibitors (62). An enrolling clinical trial in MPM patients is examining the relationship between patient genotype and response to the PARP inhibitor olaparib (NCT03531840). Another PARP inhibitor, niraparib, is being tested in patients with BAP1 and other DNA damage response (DDR) pathway deficient neoplasms including mesothelioma (NCT03207347).

BAP1 inactivation also works as a putative epigenetic regulator involved in the polycomb repressive complex 2 (PRC2) and enhancer of zeste-homolog 2 (EZH2) pathway. Mesotheliomas with BAP1 loss proved to be responsive to EZH2 inhibition *in vitro* and *in vivo* (63). EZH inhibition may then represent a promising strategy, with tazemetostat showing a promising disease control rate of 51% at 12 weeks in a multicenter phase 2 trial (64).

CDKN2A is a tumor suppressor gene frequently inactivated in mesothelioma. CDKN2A encodes the ADP-ribosylation factor (ARF, also known as p14) and INK4A (also known as p16) via alternative reading frames (65). By inhibiting cyclin–dependent kinase 4 (CDK4) and CDK6, INK4A decelerates the G1–S cell cycle transition. Small molecules CDK4 and CDK6 inhibitors induce apoptosis in CDKN2A-mutated tumors (66–69) and MPM cell lines viability was inhibited in a dose-dependent manner by the CDK4/CDK6 inhibitor abemaciclib (70). Combined with radiotherapy, this agent also completely suppressed tumor growth in a mouse model of MPM (70). These finding led to the investigation of abemaciclib in p16INK4A negative MPM patients [NCT03654833 (MiST)].

The hepatocyte growth factor (HGF), by binding to the MET receptor and activating its downstream target PI3K has been shown to enhance MPM cell proliferation, migration and invasiveness. Therefore, this pathway represents a compelling therapeutic target in this disease (71). However, the modest response rate observed in the early phase trials assessing agents targeting this pathway (72), indicates that combination regimens with other classes of antitumor agents with a sufficiently wide therapeutic window, will be necessary.

The enzyme argininosuccinate synthetase 1 (ASS1) leads to arginine biosynthesis from citrulline and is epigenetically suppressed in a high proportion of mesothelioma cell lines (73). Loss of ASS1 renders mesothelioma cells addicted to exogenous arginine (74), and this defect may be therapeutically exploited by pegylated arginine deiminase (ADI–PEG20), which works by clearing circulating arginine (73). Non-epithelioid (biphasic and sarcomatoid) MPM subtypes are characterized by a 75% rate of ASS1 loss and disease control rate (DCR) of this subgroup resulted 94% in the TRAP Phase I trial (75) of ADI-PEG 20 combined with 1st-line pemetrexed and cisplatin chemotherapy. Results from the randomized, placebo-controlled, double-blind phase 2/3 global ATOMIC-meso trial (NCT02709512) in non-epithelioid MPM are awaited.

In conclusion, despite our improved understanding of the biology of MPM, response to targeted therapies is hampered by intra-tumor heterogeneity and it is still unclear whether most of the actionable mutations constitute clonal or sub-clonal driver events. Longitudinal prospective studies, such as the TRACERx study in lung cancer (76), aiming at elucidating mechanism of resistance to treatment, are still missing in MPM. Properly designed clinical trials, which stratify patients for predictive biomarkers, are warranted. To this regard, patients enrolled in the MiST trial (NCT03654833) are currently offered a specific study treatment (either the parp-inhibitor rucaparib, the CDK4/6 inhibitor abemaciclib, the combination of the PD-1 inhibitor pembrolizumab and the AXL inhibitor bemcentinib or the combination of the PD-L1 inhibitor atezolizumab and the anti-angiogenic agent bevacizumab) determined by the results of the molecular panel testing of their diagnostic tumor block. The ones who exhibit positive testing in more than one biomarker, will potentially be eligible for a subsequent protocol upon disease progression. This trial design is aimed at providing a more tailored approach for MPM patients.

### Mesothelin Targeted Therapies

Mesothelin (MSLN) is a glycoprotein with high expression in epithelioid mesothelioma and low expression in normal tissues, thereby it represents an attractive target for several therapies. A phase II trial comparing amatuximab (an anti-MSLN chimeric monoclonal antibody) plus first-line chemotherapy vs. chemotherapy alone was prematurely stopped in January 2017, not because of unacceptable toxicity but because of business reasons (NCT02357147).

According to a public announcement, anetumab ravtansine (an antibody-drug conjugate made by combining a human anti-MSLN antibody and the maytansinoid tubulin inhibitor DM4) also failed to improve PFS compared to vinorelbine in a randomized phase II trial for patients progressing after first-line (NCT02610140) (77).

CRS-207 is a live, attenuated, non-virulent, Listeria monocytogenes (LADD) encoding human MSLN. After receiving two priming infusions of CRS-207, followed by pemetrexed/cisplatin chemotherapy, and CRS-207 booster infusions in a phase Ib trial, 89% (31/35) of patients had disease control; one complete response (3%) and 19 partial responses (54%) were reported. Reduction of tumor size was also observed post-CRS-207 infusion prior to chemotherapy in 11 patients and no treatment-related serious adverse events or deaths were observed. These results suggested that combining CRS-207 with traditional chemotherapy might potentially result in increased anti-tumor activity (78). However, after a phase II trial had showed no clinical activity of the combination of CRS-207 with PD-1 inhibition (NCT03175172), clinical development of this therapy was discontinued.

LMB-100 is a next generation immunotoxin against MSLN that consists of a humanized fragment of the anti-MSLN Fab bound to a de-immunized Pseudomonas exotoxin (PE). This PE-fusion protein has been engineered to decrease its immunogenicity. A Phase I, open-label study to investigate the safety, pharmacokinetics, and activity of LMB-100 in relapsed MPM patients is planned to complete accrual this year (NCT02798536).

Evaluating new combinations of MSLN directed therapies with checkpoint inhibitors and integrating MSLN targeting into new approaches such as adoptive T cell transfer might constitute the next step in the field, as first results have been promising (79).

### Immunotherapy

#### Immune Checkpoint Inhibitors

The immune system is known to play a key role in MPM. Immune suppression locally induced by the tumor is high (80). Survival of patients with MPM is longer when tumors are highly infiltrated by cytotoxic CD8^+^ T cells (tumor-infiltrating lymphocytes), whereas PD-L1 expression is associated with shorter survival (median OS 5.0 in patients who are PDL1-positive vs. 14·5 months PDL1-negative patients; *p* < 0.0001) (81, 82). Due to their ability to restore the capacity of immune system to counterattack tumor growth, CIs (directed toward CTLA4, PD1, PDL1 or their combinations) started to be investigated in MPM patients. A large randomized phase IIb trial, assessing tremelimumab, an anti-CTLA4 mAb, vs. placebo in a second or third-line setting did not show superiority of the immunotherapy in terms of OS (83). Looking at agents targeting the PD-1/PD-L1 pathway, interesting results were reported in the first early phase trials with overall response rates (ORR) ranging from 9 to 29% in patients previously treated with chemotherapy (84).

As shown in other types of cancer (85), combining CTLA-4 and PD-(L)1 mAb might further improve outcomes. In a single-center, single-arm, phase II trial (INITIATE) (86), the combination of ipilimumab and nivolumab for the treatment of recurrent MPM was assessed. Of the 34 patients evaluated for radiological response at 12 weeks, ten (29%) patients were partial responder and 13 (38%) had stable disease; adverse events were quite frequent (94% of patients) with 12 (34%) patients reporting grade 3 toxicity. Another randomized, non-comparative, open-label, phase 2 trial (MAPS2), conducted in 21 hospitals in France (87), met its primary endpoint of DCR after randomization in the first 108 patients. This trial aimed to assess the anti-PD1 mAb alone (nivolumab) or in combination with anti-CTLA4 (ipilimumab) mAb in MPM patients who progressed to first-line chemotherapy. Twenty-four (DCR 44%) of 54 patients treated with nivolumab and 27 (DCR 50%) of 54 patients treated with nivolumab plus ipilimumab achieved disease control at 12 weeks. Objective responses were ten (19%) with nivolumab and 15 (28%) with nivolumab plus ipilimumab. Again, the safety profile was consistent with previous data on the combination. To note, three (5%) treatment-related death were reported with the combination (one fulminant hepatitis, one encephalitis, and one acute kidney failure).

These findings confirm the promising activity of both single and double check-point blockade in MPM patients who have relapsed. However, data presented at 2019 ESMO conference from the European Thoracic Oncology Platform (ETOP 9-15) PROMISE-meso randomized phase III trial (NCT02991482) comparing PD-1 inhibition with pembrolizumab to institutional choice single agent CT (gemcitabine or vinorelbine) as second-line treatment failed to show superiority of PD-1 treatment (88). Nearly four times more patients responded to immunotherapy (ORRs were 22% with pembrolizumab vs. 6% in CT, *p* = 0.004), but these responses were not translated into delayed progression or improved survival (median PFS was 2.5 months (95% CI 2.1–4.2) with pembrolizumab and 3.4 months (95% CI 2.2-4.3) with chemotherapy, HR 1.06 (95% CI 0.73–1.53), *p* = 0.76). In this study long-term responders to pembrolizumab were also found, again underlining the importance of understanding which patients should receive this treatment instead of chemotherapy (88). Data from another randomized trial comparing nivolumab vs. placebo in patients pre-treated with at least two lines of chemotherapy [NCT03063450 (CONFIRM)], are also warranted in order to select the best strategy. At the current time, results from the MAPS2 trial supported the National Comprehensive Cancer Network (NCCN) panel decision to introduce either nivolumab or nivolumab plus ipilimumab as treatment options in relapsed MPM patients and nivolumab was approved in Japan as second-line treatment after results from a multicenter, open-label, single-arm, Japanese phase II study in MPM (MERIT) were reported, with ten (29%) patients showing an objective response (89).

Similar to other cancers, there might be a subgroup of MPM patients who might obtain a larger benefit from CIs, but relevant biomarkers have not been determined yet. Tumor PD-L1 IHC expression (with a cut-off of 1%) was correlated to ORR in both groups of MAPS-2 trial (nivolumab alone or nivolumab combined with ipilimumab) (87) but resulted in a better OS only in the nivolumab group. These correlations were not consistent in another phase II trial with nivolumab (90) and, although PD-L1 status may be associated with sensitivity to CIs, also patients with low PD-L1 expression benefit from this treatment, with a reported ORR of 11.1% (91). Intra-patient heterogeneity, different cut-points for PD-L1 positivity and lack of assay standardization also prevent PD-L1 from being used as the only selection criteria for CIs-treatment in MPM. This should lead researchers to investigate other tumor and patients' characteristics (histological subtype, performance status, blood-derived tests) to get an upfront identification of patients who are likely to respond to CIs and integration of multiple parameters (infiltration of CD8 and other subpopulations of T-cells (92), genomic signatures, specific mutations, expression of different checkpoint inhibitors) beyond PD-L1 status will be crucial.

To improve response rate to CIs in MPM patients, two options may be pursued. The first one is to move CIs toward the first-line setting, where the reinvigoration of the immune system may be stronger and more efficient, and to combine them with chemotherapy, similar to what happened in non-small cell lung cancer. Results of the addition of the PD-L1 inhibitor durvalumab to cisplatin and pemetrexed were presented in form of an abstract at the 2018 World Conference on Lung Cancer (93), showing a PFS of 6.2 months with a 48% ORR in the context of a non-randomized phase II trial—ORR is 41.3% with first-line chemotherapy alone, as historically reported (14). In the United States, a similar phase II trial investigating durvalumab (MEDI4736) in combination with chemotherapy for first-line treatment of MPM is currently in the analysis phase (NCT02899195). The addition of either pembrolizumab (NCT02784171) or nivolumab (in a Japanese population) (94) to chemotherapy is also being studied. The combination of ipilimumab and nivolumab is being compared with the cytotoxic chemotherapy standard in the first-line setting as well, with about 600 patients expected to be enrolled in a phase III trial (95).

The second option may be to combine CIs with either different immune-modulatory molecules, targeted therapies, antiangiogenic agents, or radiotherapy. Additional co-inhibitory and co-stimulatory molecules such as T-cell immunoglobulin and mucin-domain containing-3 (TIM3, also known as HAVCR2), lymphocyte activation gene 3 (LAG3) and inducible T cell co-stimulator (ICOS) are being investigated in mesothelioma (96–98). Inhibiting FAK together with PD-1, may enhance immune cell-associated antitumor cytotoxicity *in vivo*, which is hampered by expression of PD-L1 (99) and this represented the rationale for a phase I/IIa currently ongoing (NCT02758587). Similarly, in addition to the direct anti-tumor effects, pegylated arginine deiminase (ADI-PEG 20) may boost tumor immune surveillance and might be a good primer for an additional anti-tumor immune therapy (100), raising the question whether combining ADI-PEG 20 with PD-1/PD-L1 blockers may further enhance these drugs' anti-tumor efficacy (101).

Early phase trials also assessed the combination of anti-PD1/PDL1 agents and MSLN-directed therapies (in MSLN-positive patients). After results from a pre-clinical murine lung tumor model (CT26hMeso) demonstrated anti-PD1 enhanced LADD-induced tumor response (102), a phase 2 single-arm study of CRS-207 with pembrolizumab in relapsed MPM was started but no responses were showed, and the study was discontinued (102). Two other phase 2 trials (NCT03644550, NCT03126630) assessing the combination of pembrolizumab with the anti-MSLN Immunotoxin LMB-100 and with the antibody-drug conjugate anetumab ravtansine are currently enrolling patients, with the latter one also randomizing patients to pembrolizumab alone as active comparator.

Growing evidence that pro-angiogenesis factors have immunosuppressive activity has led researchers to evaluate the potentially synergistic combination of antiangiogenic agents and immunotherapy also in the treatment of MPM. VEGF signaling has been shown to attenuate the immune antitumor response by either influencing lymphocyte trafficking across endothelia to the tumor or directly inducing inhibitory immune cell subsets (103). Several trials are aiming to address whether the combination of CIs and antiangiogenic agents (either mAbs as bevacizumab and ramcirumab or TKIs as nintedanib) is able to improve outcomes in MPM patients (NCT03762018, NCT02856425, NCT03502746).

Finally, similarly to certain types of chemotherapy, radiotherapy can be exploited for its ability to cause immunogenic cell death (ICD), thus priming the release of damage-associated molecular patterns (DAMPs) and tumor-associated antigens (TAAs) and inducing a systemic anti-tumor immune response, that may be further enhanced by PD-1 (pembrolizumab) or PD-L1 (avelumab) blockade (NCT02959463, NCT03399552).

### Vaccines

Vaccines represent another way to boost the immune system activation against the tumor. Both protein, vector and cell-based vaccines have been tested in MPM.

Galinpepimut-S is a WT-1 synthetic peptide vaccine made out of molecules similar to those in the WT1 protein. After a phase II trial confirmed vaccine's safety when administered in the adjuvant setting, researchers' efforts are currently directed toward the assessment of the combination of galinpepimut-S and nivolumab (NCT04040231). It has been hypothesized that the negative influence of tumor microenvironment factors on the immune response might be mitigated by nivolumab, thus providing the opportunity for the reinvigorated immune cells, specifically sensitized against WT1 by the vaccine, to invade and destroy cancerous growth deposits.

Dendritic cells are antigen-presenting cells that present tumor-associated antigens (TAAs) to the immune system by trafficking from tumors to lymph nodes. They are essential in priming proliferation and activation of CD8^+^ cytotoxic T–lymphocytes and CD4^+^ helper T-lymphocytes resulting in a potent and specific anti-tumor response (104). Dendritic cell function is hampered in cancer patients by tumor-derived soluble factors that suppress their immune-stimulatory ability (105, 106). However, dendritic cells can be generated in large amounts *ex vivo* and loaded with TAAs, prompting their recent usage as cancer vaccines in several neoplasms, including MPM. Several sources of tumor antigens (mRNA, peptides, proteins or whole tumor cell lysate) can be used to load DCs (107). Because TAAs are difficult to identify in mesothelioma (thus excluding peptides as best source), and adequate tumor tissue is rarely obtained from mesothelioma patients (108, 109), an allogenic tumor lysate has been developed (110). Results from a first-in-human clinical trial involving nine MPM (non-progressive after at least 4 cycles of chemotherapy) showed that this approach is safe (no dose-limiting toxicities were established) and led to radiological responses and promising survival data, with median PFS of 8.8 months and median OS not reached (110). A large multicentric phase II/III randomized trial with allogeneic-lysate pulsed dendritic cell immunotherapy as maintenance treatment after platinum-based chemotherapy is currently enrolling in Europe [NCT03610360 (DENIM)] (111).

### T Cell Therapies

Another promising cell-based strategy in mesothelioma is represented by adoptive T cell therapy. Data from a phase I trial investigating chimeric antigen receptor (CAR) T cell therapy targeted to the MSLN protein in 19 MPM patients progressed following standard platinum-based chemotherapy were recently reported (79). A single-dose of second-generation CD28-costimulated MSLN-CAR T cells with the Icaspase-9 safety gene (IcasM28z) was given intrapleurally (as recommended by previous observations in murine models, in which intrapleural administration vastly outperformed intravenous infusion) (112) with or without cyclophosphamide preconditioning. No evidence of on-target, off-tumor or therapy related toxicity was seen, and CAR T-cell persistence was associated with decreased levels of serum soluble MSLN-related peptide (SMRP) levels (>50% compared to pretreatment) and evidence of tumor response. Of the 14 patients who received anti-PD1 agents, off-protocol, after the CAR T-cell therapy, 2 achieved a complete metabolic response, 5 obtained a partial response, and 4 had stable disease. Combining anti-PD1 therapy with CAR T cells is also supported by prior preclinical data showing that CAR T cells become functionally exhausted in the presence of a large tumor burden and that anti-PD-1 therapy can reactivate these exhausted cells (113).

### Virotherapy

Oncolytic viral therapy represented in the last decades an emerging field of immunotherapy and a promising experimental strategy. Viruses can act by infecting cancer cells and leading to cell lysis after replication. This renders tumor-associated and viral antigens recognizable to the immune system, thus triggering antitumor immune responses (viroimmunotherapy) (114, 115). Oncolytic viruses need also to be tumor selective, and although malignant cell-specific oncolysis naturally occurs because of the impairment of the type I interferon pathway in many tumor cells, viruses may be engineered in order to increase their selectivity. Viruses may be used also for gene therapy, thereby therapeutically changing the infected tumor cells by gene transfer (116).

The pleural location and the peculiar pattern of growth (mostly localized), which provide access to direct intratumoral injection of virus, make MPM an ideal candidate for assessing the efficacy of oncolysis (116). The safety of virotherapy has been assessed and some clinical response have been reported (114). Among the many viral vectors that have been investigated, the recombinant replication incompetent adenoviral (ADV) vector encoding human interferon-α (IFNα, a naturally-occurring protein with anti-cancer properties) administered “*in situ*” (intrapleurally) with celecoxib (to reduce the number of immunosuppressive MDSCs) before chemotherapy, was well tolerated and appeared to improve overall survival rates (117). Combinations of virotherapy with CIs, chemotherapy, and radiation are expected to further boost the effects on antitumor immunity and represent the object of ongoing trials (118–120), such as the phase III INFINITE trial (NCT03710876), in which about 300 patients will receive gemcitabine and celecoxib with or without the ADV-delivered IFNα-2b (rAd-IFN).

## Conclusion

In the past two decades there was limited success in the development of novel therapies for MPM. Multiple biases in the design of clinical trials and the peculiar biological features of MPM were most probably responsible for delaying the discovery of effective therapeutic agents. Most of the previous trials attempted to readapt drugs that succeeded in other cancer types to MPM. However, they were either too small or not stratified for predictive biomarkers. Results from phase II studies were often not replicated in larger, randomized, phase III trials, pointing out that well controlled trials with appropriate size and duration are crucial to confirm the efficacy of a new agent (121).

In the last few years, mesothelioma genetics, epigenetics, and the tumor microenvironment (especially immune-biology) have been studied more deeply and this knowledge has started to be properly applied to discover new therapies. In particular, expectations are now high that CIs and other immunotherapies will have a leading role in the future therapeutic armamentarium of MPM. Noteworthy, scientific evidence supporting the use of CIs in MPM are still incomplete, mainly based on non-randomized studies with surrogate end-points and they have not been always replicated in the real-life context. Because of the risk of cumulative toxicities and of the high cost of these drugs (especially of combinations), validated biomarkers are urgently needed to select MPM patients who may benefit from immunotherapies. Since the “one-size fits all” approach is not recommended for immunotherapy and MPM and the efficacy of CIs is still to be established in a larger population, there is still a need for new treatments in MPM and the implementation of other targeted agents is eagerly awaited.

Only a close collaboration between medical centers and industry may lead to the conduction of well-designed, biomarker-driven clinical trials. New trials should always include translational and quality of life components, in order to clarify the molecular basis of response or progression to treatments and to finally improve the degree of reliability of the possible benefit of new therapies for MPM.

## Author Contributions

LC and JA wrote the manuscript and generated the figure and table. RH and DS contributed to the revisions of the manuscript. All authors approved the manuscript for publication.

### Conflict of Interest

JA: No relationship to disclose in relation to the submitted work. Relevant financial activities outside the submitted work: Stock or Other Ownership: Amphera Consulting or Advisory Role: Bristol-Myers Squibb, MSD Oncology, Boehringer Ingelheim, Eli-Lilly, Roche Speakers Bureau: AstraZeneca. Research Funding: Genentech (Inst), Boehirnger Ingelheim (inst). Patents, Royalties, Other Intellectual Property: Patent: Tumor cell lysate for dendritic cell loading (Inst), SNP analyses for immunotherapy (Inst). The remaining authors declare that the research was conducted in the absence of any commercial or financial relationships that could be construed as a potential conflict of interest.
